# Understanding Teacher Self-Efficacy to Address Students’ Social-Emotional Needs in the COVID-19 Pandemic

**DOI:** 10.1177/00420859221099834

**Published:** 2022-05-20

**Authors:** Cassandra R. Davis, Courtney N. Baker, Jacqueline Osborn, Stacy Overstreet, the New Orleans Trauma-Informed Schools Learning Collaborative

**Affiliations:** 1 Department of Public Policy, 2331 The University of North Carolina at Chapel Hill, Chapel Hill, NC, USA; 2 Department of Psychology, 5783 Tulane University, New Orleans, LA, USA

**Keywords:** Coronavirus pandemic, teacher self-efficacy, race, urban schools

## Abstract

Teachers are returning to schools during the COVID-19 pandemic under the weight of unprecedented stressors to engage a student body that has also experienced stress and trauma. In this study, we examined how confident 454 teachers (55% Black) from 41 charter schools in New Orleans, Louisiana, were in their ability to address students’ social-emotional needs upon their return to school. Results showed that Black teachers were more likely to report a greater sense of efficacy in addressing students’ needs. Both Black and White teachers identified the top three resources needed to assist students: mental health supports, trainings, and in-class resources.

## Introduction

The widespread and significant emotional toll of COVID-19-related stressors on students became clear in the first few months of the pandemic. As early as May 2020, nearly 30% of U.S. parents reported that their children had experienced harm to their mental health due to COVID-19 (U.S. Department of Education, 2021). The impact was even greater for students of color. Pre-pandemic disparities in educational resources, opportunities, and outcomes experienced by many students of color, combined with the disproportionate impacts of COVID-19 on Black and Latinx families, deepened longstanding inequalities in access to quality education and mental health services (U.S. Department of Education, 2021). Before COVID-19, students of color were more likely than their White peers to attend schools in high poverty communities that had access to fewer resources, less experienced teachers, and produced lower standardized test scores (Bettini & Park, 2021; Milner, 2013). During the pandemic, Black students were more likely to lose a parent or caregiver to COVID-19 (U.S. Department of Education, 2021) and they, themselves, were far more likely to be hospitalized or to die due to COVID-19 than their White peers (Bixler et al., 2020). Students of color were also more likely to reside in households with economic insecurity and a lack of technology access that challenged online learning during the pandemic (U.S. Department of Education, 2021).

As early as the Spring of 2020, principals and teachers were already anticipating the immense student mental health needs that would require attention before students could resume learning when they returned to school in the Fall of 2020 (Hamilton et al., 2020). In particular, principals whose student body was primarily students of color were more likely to identify supports for student social-emotional learning as a significant need compared to those leading majority-White schools. In the face of disasters, teachers often serve as one of those supports.

Teachers can play a crucial role in helping students feel safe, supported, and connected by creating affirming learning environments and positive student relationships. For students of color in urban communities, affirming practices and positive teacher-student relationships can help students cope with personal stressors and serve as a buffer against systemic racism and discrimination (Ladson-Billings, 1994; Sosa & Gomez, 2012). Unfortunately, the unprecedented nature of the COVID-19 pandemic and the resulting personal and professional challenges faced by teachers in urban schools (Baker et al., 2021) may challenge their self-efficacy to support students of color whom the pandemic has most impacted. Teaching self-efficacy is a multidimensional construct that includes instructional efficacy as well as efficacy in managing classroom behaviors, providing emotional support, and adapting to change (Tschannen-Moran & Woolfolk Hoy, 2001). Teacher self-efficacy is critical to supporting student psychological health and emotional safety as well as fostering student learning (Sosa & Gomez, 2012).

Teacher self-efficacy, specifically classroom management self-efficacy, is also vital for teacher functioning and well-being (Jennings & Greenberg, 2009). In the immediate aftermath of pandemic-related school closures, teacher perceptions of their online classroom management skills were associated with better coping, more job satisfaction, and lower levels of depression and anxiety (Herman et al., 2021). The goal of the current study is to understand how teachers think about their classroom management self-efficacy as they look toward the reopening of schools. In addition, the current study examines individual and contextual factors associated with teacher self-efficacy to identify ways to boost their confidence, support their well-being, and enhance their ability to help their students.

This mixed-methods study was conducted within a high-poverty urban school district primarily serving Black students to address the following questions:

1. Do teachers feel efficacious in addressing the social and emotional needs of students of color stemming from experiences of pandemic-related stress and trauma?
2. What factors influence teacher self-efficacy? And,
3. What resources do teachers believe they need to support their students?

Study findings have the potential to contribute critical new insights related to urban education in the context of disasters. Trends on increased urbanization show that a greater concentration of educators, students, and their families reside in smaller spaces, leaving them more exposed to future hazards (Skidmore & Lim, 2020). As COVID-19 continues to evolve and climate change continues to disrupt life, urban pedagogy will need to include discussions around disaster mitigation, preparation, and recovery, specifically for spatially located schools in at-risk areas. Understanding the factors that influence teacher self-efficacy in disasters will be essential to helping schools support the needs of their teachers and their students.

### Literature Review Supporting Conceptual Frameworks

The conceptual frameworks of teacher self-efficacy and racial congruence are integrated into the following literature review to understand what it means for teachers to be confident in supporting students of color during a pandemic and how much of that confidence depends on racial congruence with their students. Bandura’s (1997) original conceptualization of sources of teacher self-efficacy included two factors of importance in the current study: physiological and emotional states and social persuasion (Milner & Woolfolk Hoy, 2003). However, his original conceptualization did not fully consider how racial congruence between teachers and students might influence their self-efficacy (Milner & Woolfolk Hoy, 2003; Siwatu et al., 2011). Teacher-student racial congruence refers to students’ racial and ethnic composition in relation to the teacher's own race/ethnicity (McCarthy et al., 2020). Racial congruence has been associated with teacher perceptions of student behavior and teacher classroom management self-efficacy (Geerlings et al., 2018; Kunemund et al., 2020). Each of the factors associated with teacher self-efficacy is considered in the literature review.

The physiological and emotional arousal associated with the emotional distress brought on by the pandemic could influence whether teachers feel prepared to provide emotional support to their students. When a teacher's mental health is threatened due to personal- or work-related stressors, they may lack sensitivity to student needs, be more likely to disengage and withdraw from their students, have difficulty making effective changes to classroom management practices to address emerging student needs and be more likely to employ exclusionary discipline practices (Jennings & Greenberg, 2009). Unfortunately, we know that teachers are returning to school under the heavy weight of their pandemic-related stressors (Baker et al., 2021; Cipriano & Brackett, 2020), including separation from family and friends, increased workloads, and difficulties in the transition to working from home. Teachers have also experienced indirect exposure to trauma through their increased awareness of student struggles and through the learning of deaths in the families of their students. Not surprisingly, the pandemic-related stressors that teachers have experienced have harmed their mental health and coping (Baker et al., 2021). As teachers struggle to manage their pandemic-related challenges to mental health, their sense of efficacy in addressing student needs related to similar experiences is likely to be negatively impacted (Jennings & Greenberg, 2009).

There is little work that explores the extent teachers recover from and are supported through a disaster. Even when looking at the costliest storms, such as Hurricanes Katrina (2005) and Harvey (2017), few studies followed teachers’ recovery in the aftermath of the event (Davis et al., 2021). In one study, authors Seyle et al. (2013) created an intervention to support teachers after an earthquake. Their work found that teachers who did not use the intervention were more likely to suffer from burnout and exhibit lower self-efficacy levels than their colleagues who did not use the service.

In addition to the role of emotional arousal discussed above, teacher confidence in system-level supports responsive to their needs is critical for teacher efficacy. System-level supports can be viewed as one aspect of social persuasion that influences teacher self-efficacy (Milner & Woolfolk Hoy, 2003). A lack of sufficient resources, training, and administrative and peer support can increase demands on teachers and challenge their sense of efficacy (Hoy et al., 1990). Given the unprecedented demands schools face with ensuring physical safety and accelerating student learning, teachers have a legitimate reason to doubt whether their needs will be prioritized when schools reopen (Modan, 2020). Experts are concerned that schools will not have the means or motivation to reopen in ways that prioritize teacher training and preparation for emotional support and mental health (U.S. Department of Education, 2021). All of these factors may undermine teacher confidence in their ability to address the extraordinary social and emotional needs of their students.

Another factor that could influence teacher self-efficacy is a lack of racial congruence between the teachers and students (Bentley-Edwards et al., 2020; Kunemund et al., 2020; McCarthy et al., 2020). Racial congruence refers to school contexts where teachers share the same racial identity as most of their students (Martinez, 2020). When teachers experience racial congruence with their students, they may be more likely to adopt culturally sustaining pedagogical techniques, which Paris and Alim (2017) described as drawing on “dynamic cultural dexterity” to address the evolving social needs of students through asset-based approaches that value students’ cultural ways of being (p.1).

Scholars have argued that Black teachers have an increased ability to understand the cultural context of Black students’ lives, resulting in greater efficacy in understanding and responding to their needs (Lindsay & Hart, 2017; Villegas & Irvine, 2010). As Milner (2006) stated, Black teachers can maintain “culturally informed relationships” and “cultural connections” with their Black students that allow for a fruitful classroom environment (p.10). Specifically, Black women teachers use their intersectional experiences through race, class, and gender to inform their pedological practices within the classroom that create inclusive space for all students (Muhammad et al., 2020). Black teachers often take on the role of an academic othermother- a term that describes the identity of a Black female teacher who represents a mother figure within the school (Greene, 2020). These othermothers inherently focus on the whole child by meeting their academic, physical, and emotional needs. Black teachers have related their role of teaching to nurturing their mother's gardens by connecting their profession to a spiritual mission of supporting and uplifting children of color (Dixson & Dingus, 2008).

Where Black teachers can connect with Black students around evolving and challenging situations, White teachers can experience racial stress in their interactions with Black students due to the lack of a culturally shared knowledge base (Bentley-Edwards et al., 2020; McCarthy et al., 2020; Warren, 2015). Teachers’ ability to provide culturally relevant emotional support may decrease with little shared knowledge (Pollack, 2013). Evidence of the importance of teacher-student congruence for teacher efficacy is provided by Geerlings et al. (2018), who found that when there was ethnic incongruence between teachers and their students, teachers felt less efficacious in supporting student engagement and in working with students experiencing high internalizing behaviors.

Another example provided by Gilliam et al. (2016) showed that when teachers were provided with information about the familial stressors of students, they rated child behavior as less severe when there was a teacher-student match in terms of race and more severe when there was not. When teachers and students are of the same race, the authors speculated that information about familial stressors might engender greater empathy and contribute to a greater sense of efficacy in responding to student needs. When there is no match, information about familiar stressors may lead teachers to feel a sense of hopelessness in their ability to respond to student needs, decreasing their sense of efficacy.

The heightened awareness of racial disparities and systemic racism that teachers have reported experiencing during the pandemic can amplify racial stress and further deteriorate White teachers’ sense of efficacy to engage and support Black students (Bentley-Edwards et al., 2020). Taken together, these findings suggest that teacher-student racial congruence may have a powerful influence on teachers’ sense of efficacy in addressing the pandemic-related social and emotional needs of students of color.

### Current Study

As with other disasters, teachers will play an essential role in addressing students’ social and emotional needs amid the COVID-19 pandemic (Barrett et al., 2012; Cannon et al., 2020). We examined teachers’ sense of efficacy to take on this task and asked them what resources they would need to be effective. Based on our conceptual frameworks of teacher self-efficacy and racial congruence, we hypothesized that teachers who reported experiencing more stressors, worse mental health, and lower confidence in system-level supports would report lower efficacy ratings. We also hypothesized that Black teachers in our sample of urban schools serving predominately Black students would report higher efficacy ratings than White teachers. A qualitative analysis of open-ended responses describes the resources teachers felt they would need to adequately address the social and emotional needs of their students.

## Methods

### Site of the Study

The study took place in New Orleans, Louisiana. Based on Milner’s (2012) assessment of urbanicity, New Orleans is classified as a large city or urban emergent location, not a major city or an urban intensive environment (e.g., New York). Urban emergent sites have similar characteristics as urban intensive sites related to limited resources, teacher quality, and student academic outcomes.

At the start of the pandemic, New Orleans had the fastest growth rate of COVID-19 cases in the world during the 13 days following the first confirmed case (Silverman, 2020). All schools in the city were closed by government mandate on March 13, 2020, and remained closed for six months before reopening for the 2020–21 school year in mid-September. New Orleans is a portfolio school district comprised entirely of independent charter schools. Eighty-four percent of New Orleans public school students live in poverty and 81.3% are Black (New Orleans Education Equity Index, 2017). The data utilized in the current study were gathered as part of a needs assessment conducted in April-May 2020.

Unfortunately, New Orleans’ schools and teachers are no stranger to facing major environmental disruptions. In August 2005, Hurricane Katrina devastated the city and became the nation's costliest catastrophe in history, with an estimated $161 billion in damage (National Oceanic and Atmospheric Administration, n.d). With the city's school system in ruins, charter schools and teacher organizations like Teach for America, New Teacher Project, and New Leaders for New Orleans moved in to take over schooling (Lincove et al., 2018). Research showed that during this time, teachers impacted by Hurricane Katrina expressed having low levels of self-efficacy in reaching their students’ needs and deemed environmental factors as the reason for shortcomings in the classroom (Daniel & Harwell, 2010). With a repeated and large-scale disaster, teachers are yet again facing their own trauma and challenges to their efficacy in supporting the academic and emotional needs of students across New Orleans.

### Participants

Four hundred and fifty-four teachers from 41 public charter schools in Orleans Parish completed the survey. Respondents represented about 14.5% of the total population of teachers and 48% of New Orleans public schools (Babineau et al., 2020). Teachers from five schools comprised about half of the sample, teachers from another eight schools included 30% of the sample, and teachers from the remaining 28 schools comprised 20%. Half of the sample (51.5%) reported teaching elementary school, 23.6% taught middle school, 15.4% taught in high school, and 10.5% reported having roles that crossed grade levels.

The study sample is similar to New Orleans public school teachers (Table 1). As illustrated in Table 1, there was an insufficient representation of teachers from racial/ethnic groups other than Black and White groups. Given that our hypothesis about race's influence on teacher efficacy is based largely on studies examining differences between White and Black teachers, we limited the study sample to the 390 participants who identified as either Black or White.

**Table 1.** Sample Comparison to New Orleans Teacher Profile (in Percentages).

	Sample Profile	New Orleans Teacher Profile*^a^*
Race*^b^*		
Black	54.5	53.4
White	31.9	39.4
Latinx	2.7	4.6
Other	10.9	2.6
Gender*^c^*		
Female	81.5	73.5
Male	18.5	26.5
Experience		
0 – 5 years	51.2	50.5
6 – 10 years	21.1	21.0
11 – 15 years	11.6	11.6
16 + years	16.2	16.9

^a^ Based on data provided by the State of Public Education in New Orleans 2019–2020 report (Babineau et al., 2020).

^b^ Race categories are limited to those tracked by Babineau et al. (2020).

^c^ The study sample was similar to the population of New Orleans public school teachers with the exception of gender; male teachers are somewhat underrepresented in our sample.

### Procedure

The current study used data gathered from a local needs assessment conducted by the New Orleans Trauma-Informed Schools Learning Collaborative. The needs assessment survey was designed to understand the impact of the COVID-19 pandemic on teachers, their school community, and their teaching. Additionally, the survey gathered data directly from teachers about their immediate needs to support well-being and remote instruction, as well as the longer-term needs to prepare for school re-openings. Findings related to the prevalence and impact of pandemic-related stressors and the needs of teachers early in the pandemic are reported by Baker et al. (2021). The current study utilized quantitative survey items most relevant for understanding teacher self-efficacy as well as one qualitative item that asked teachers to describe what they would need to meet their students’ social-emotional needs effectively (e.g., supports for self-efficacy). Two other qualitative items related to system-level efficacy were not included in the current study due to space limitations and less direct relevance to the study questions.

An anonymous online survey using Qualtrics was open to New Orleans area teachers from April 30 to May 15, 2020. Teachers were invited to complete the survey through direct invitations from school leaders, local listservs and organizational newsletters, social media, and word of mouth. The Institutional Review Board determined that using the deidentified needs assessment data did not qualify as human subjects research (#2020-1416). Since the needs assessment took place under the high demands of moving all instruction to virtual formats, the survey was designed to assess essential domains of experience and functioning in the most efficient way possible. Single-item indicators and shortened versions of existing surveys were used to maintain the brevity of the survey, reduce participant burden, and support survey completion (Donnellan et al., 2006; Hoerger, 2010). Research in mental health and well-being in the workplace, including research conducted with teachers (Eddy et al., 2020), has demonstrated the reliability and validity of single-item measures, which support the application of research to practical settings (Ahmad et al., 2014). Quantitative and qualitative data were gathered simultaneously. The quantitative data collection aimed to characterize the experiences and perspectives of teachers during the pandemic, and the qualitative data collection was intended to provide a deeper understanding of the quantitative findings (Palinkas et al., 2011).

### Measures

#### Demographic questionnaire

Participants’ gender, race, and age were self-reported on the survey. We also gathered employment information, including grade level taught, primary role (general education vs. special education), years of experience in the current role, years of experience at the existing school, and school name.

#### Teacher efficacy

Teachers were asked to rate how confident they were in being adequately prepared to address the stress and trauma students likely experienced during the pandemic once schools reopened. Responses were provided on a 4-point scale (1 = not at all confident to 4 = very confident). Psychometric information is not available as this item was created for the needs assessment survey. In a study of single-item measures examining well-being in the workplace, Williams (2012) found that a single-item measure of self-efficacy (I am confident in my ability to solve problems that I might face in life) was significantly correlated with the multi-item General Self-Efficacy Scale. In addition, Williams and Smith (2016) found that the same single-item measure of self-efficacy was significantly negatively associated with depression, anxiety, and negative affect.

Conceptually, the item used in the current study is most closely related to the teacher efficacy domain of emotional support developed by Zee and colleagues (Zee et al., 2016). Emotional support efficacy refers to how well teachers believe they can establish caring relationships with students and create settings in which students feel free to explore and learn, as indicated by the sample item, “How well can you establish a safe and secure environment for this student?”

#### Stressors

The Epidemic-Pandemic Impacts Inventory (EPII; Grasso et al., 2020) was adapted to evaluate the negative impact of the COVID-19 pandemic. The original scale includes 73 items within nine stressor domains related to work, education, and economics; home life and social activities; emotional and physical health; and quarantine and infection experiences. We selected 13 items that spanned the original domains (e.g., increased workload or work responsibilities, separation from family or close friends, death of a close friend or family member from this disease) in the interest of brevity. We adapted three infection-related items to assess experiences with COVID-19 within the school community (e.g., death within the families of students from this disease), one item to reflect a work-related stressor local teachers had been reporting (e.g., more acute awareness of stressors students face at home), and one item related to emotional and physical health (e.g., felt unsafe).

Participants indicated whether they had experienced a change in each of 18 stressors since the pandemic began (no = 0, yes = 1). Example stressors included “an increase in workload or work responsibilities” and “medical treatment due to severe symptoms of this disease.” Items were summed, with a possible range of 0 to 18 and an observed range of 0 to 15. Detailed information on the frequency of COVID-19 stressors within the current sample is provided by Baker and colleagues (Baker et al., 2021).

#### Mental health

Teachers responded to a single-item indicator of their mental health: “How would you rate your overall mental health since the coronavirus disease pandemic?” on a 5-point scale (1 = poor; 5 = excellent). Single-item indicators of self-rated mental health correlate with longer measures of mental health and meaningfully predict a host of indicators of stress, health, and well-being (Ahmad et al., 2014). In a longitudinal study using a community sample, Hoff et al. (1997) found that individuals who rated their mental health as poor on a single-item indicator were 4.5 and 9.97 times more likely to experience a major depressive episode in the next year than those who rated their mental health as fair or excellent, respectively. Finally, in a study utilizing the current sample, Baker et al. (2021) found that teacher ratings on the single-item indicator of mental health were associated in the expected, negative direction with COVID-19-related stressors and difficulty coping.

#### Teacher confidence in system-level supports

Teachers were asked to rate how confident they were that their school would address the stress and trauma teachers likely experienced during the pandemic once schools reopened. Responses were provided on a 4-point scale (1 = not at all confident; 4 = very confident); higher scores indicated greater confidence.

#### Qualitative items

After completing the item assessing self-efficacy, teachers were asked to respond to the question: “What resources/supports would you need to be adequately prepared to address the stress and trauma of your students?”

### Analytic Approach

#### Quantitative

Univariate and bivariate statistics were used to characterize demographic and study variables and the simple relationships between them (see Table 2). Mann-Whitney *U* tests were used to evaluate differences between Black and White teachers on self-efficacy. Bivariate relationships were calculated using Spearman's rho rank correlations for analyses with self-efficacy, mental health, and confidence in system-level supports and Pearson product-moment correlations for stressors (Xu et al., 2013). An ordinal regression analysis was fit, predicting teacher efficacy from age, race, stressors, mental health, and teacher confidence in system-level supports. All analyses were completed using SPSS Version 26.

**Table 2.** Descriptive Statistics and Intercorrelations between Study Variables.

	1	2	3	4	5	6	7	8	9	10	11
1. Age	–	−.09	−.33**	−.11*	.01	.32**	.57**	.25**	−.05	.30**	.21**
2. Gender (1 = Female; 2 = Male)	–	–	.04	.23**	−.03	−.01	−.11*	.02	−.05	.05	.08
3. Race (1 = Black; 2 = White)	–	–	–	.17**	.05	−.04	−.15**	−.31**	−.08	−.23**	−.22**
4. Grade level (1 = Elementary; 2 = Middle; 3 = High)	–	–	–	–	.07	−.16**	−.12*	−.03	−.02	−.03	.01
5. Primary role (1 = General Ed; 2 = Special Ed)	–	–	–	–	–	−.12*	−.14**	.05	−.11*	.01	.04
6. Years in current school	–	–	–	–	–	–	.48**	.02	.02	.01	−.00
7. Years in current role	–	–	–	–	–	–	–	.07	.08	.10	−.03
8. Teacher efficacy (*M* = 2.33; *SD* = .94)	–	–	–	–	–	–	–	–	−.14**	.34**	.57**
9. Stressors (*M* = 7.43; *SD* = 2.84)	–	–	–	–	–	–	–	–	–	−.31**	−.15**
10. Mental health (*M* = 2.81; *SD* = 1.03)	–	–	–	–	–	–	–	–	–	–	.27**
11. Confidence in system-level supports (*M* = 2.36; *SD* = 1.00)	–	–	–	–	–	–	–	–	–	–	–

*Note.* Relationships with teacher efficacy, mental health, and confidence in system-level supports are estimated using Spearman's rho rank correlations; relationships with stressors are estimated using Pearson product-moment correlations. *p < .05, **p < .01.

#### Qualitative open-ended responses

Open-ended responses were coded in Microsoft Excel and were organized by race (Black respondents = 238; White respondents = 138) and efficacy. Efficacy was determined using the same strategy indicated in the quantitative analysis, where teachers selected their confidence level in their ability to address the stress and trauma students likely experienced during the pandemic. The first author then exercised open-coding separately for Black and White teachers, but not by efficacy level. This form of coding allowed the qualitative researcher to examine the data, identify concepts, and categorize topics into related ideas (Strauss & Corbin, 1990). Complex responses were broken down into distinct text segments, and those text segments were individually coded. Thus, the sum of total codes is greater than the total number of responses. Coded segments were grouped into themes based on commonality and tallied to determine the frequency of each theme. Through this process, we assessed the most common themes by race and efficacy level.

The researchers used a *codebook* and inter-rater reliability checks to maintain the reliability of qualitative responses. A *codebook* is a document that holds the agreed-upon definitions of codes and themes on a given project. Researchers refer to their codebook to ensure they are coding content consistently. The codebook housed nine themes that represented content across 33 codes within this study.

Once the first author identified codes and themes, 25% of responses from Black and White participants were reviewed by the corresponding author to formalize the operational definitions of the themes and codes. The corresponding authored reviewed text to ensure that all themes and codes were relevant and that additional items were not needed. Inter-rater reliability was calculated for percent agreement for the total number of text segments within each response and percent agreement for theme assignment for those segments identified by both coding team members.

The initial agreement rate for the *total number of text segments* within each response was 68.1% for responses provided by Black participants and 62.8% for responses provided by White participants. The initial rate of agreement for *the assignment of text segments* to themes was 87.5% for responses provided by Black participants and 81.1% for responses provided by White participants. Discrepancies were discussed and resolved at a rate of agreement of 100% for both coding elements. The codebook was revised as necessary to reflect the resolution of discrepancies. After these changes, inter-rater reliability was calculated on a second set of 20 responses from Black and White participants. Inter-rater reliability was acceptable (Miles & Huberman, 1994). Agreement for the total number of text segments within each response was 85.0% within the Black sample and 75.0% within the White sample; agreement for assignment of text segments to themes was 89.9% within the Black sample and 87.5% within the White sample. Discrepancies were discussed and resolved at a rate of 100% agreement.

## Results

Eighteen participants had at least one instance of missing data, representing.02% of the total possible number of item responses. Missing data were determined to be missing completely at random, as chi-square tests of independence indicated that missingness was not related to gender, race, age, grade level taught, time at the school, or time in their teaching role. Missing data were handled using pairwise deletion.

### Research Question 1: Do Teachers Feel Efficacious in Addressing the Social and Emotional Needs of Students of Color Stemming from Experiences of Pandemic-Related Stress and Trauma?

As previous literature shows, teacher self-efficacy is vital to supporting students’ overall emotional and psychological well-being and promoting healthy student learning environments (Sosa & Gomez, 2012). Our results showed that on average, teachers were somewhat to most confident (*M* = 2.33, *SD* = 0.94) in their efficacy to address the pandemic-related stress and trauma of their students. More specifically, 14% of teachers felt very confident, 23% mostly confident, 45% somewhat confident, and 18% not at all confident to support the social and emotional needs of their students.

### Research Question 2: What Factors Influence Teacher Self-Efficacy?

Given our use of the frameworks around self-efficacy and racial congruency, we hypothesized that White teachers – those less likely to be racially congruent to their students – and teachers who reported experiencing more stressors, worse mental health, and lower confidence in system-level supports would report lower efficacy ratings. As expected, Black teachers reported a greater sense of efficacy (*M* = 2.56, *SD* = 0.99) than White teachers (*M* = 1.94, *SD* = 0.68; *U* = 10672.00, *p* < .001). Among Black teachers, 48.6% were mostly to very confident in their ability to address the social-emotional needs of their students compared to just 17.6% of White teachers.

The remaining three factors examined for their influence on self-efficacy were correlated with each other. The experience of COVID-19-related stressors was associated with poorer mental health and less confidence in the school to address teacher needs; poorer mental health was also related to less confidence in the school to address teacher needs. As hypothesized, fewer stressors, more positive mental health, and a greater sense of confidence that schools would address teachers’ needs related to their experiences with pandemic-related stress and trauma were significantly associated with a greater sense of efficacy (see Table 2 for correlations between variables).

An ordinal regression analysis was conducted to examine the relative contribution of teacher race, stressors, mental health, and system-level supports in predicting teacher efficacy (see Table 3). Although teachers’ age was significantly associated with several study variables, when age was used as a covariate in the regression analysis, it did not emerge as a significant predictor of teacher efficacy. Results provided support for the hypothesis that Black teachers would report a greater sense of efficacy in their ability to address the needs of their students related to their experiences with stress and trauma. Black teachers rated their self-efficacy almost a full standard deviation higher than White teachers (*b* = −.78, *p* < .01). In addition, teachers who reported more positive mental health reported a greater sense of efficacy (*b* = .43, *p* < .01), such that every 1-point increase on the mental health rating scale was associated with about a half a standard deviation increase in self-efficacy. Greater confidence in system-level supports for their own needs was the strongest predictor of self-efficacy to address student needs (*b* = 1.27, *p* < .01); every 1-point increase in teacher confidence in system supports was associated with about a 1.3 standard deviation increase in the self-efficacy scale. When considered simultaneously with teacher race, mental health, and system-level supports, pandemic-related stressors were not associated with teacher self-efficacy.

**Table 3.** Predicting Teacher Efficacy from Stressors, Mental Health, Race and Confidence in System-Level Supports.

	Teacher Efficacy	
	*b*	Wald *X^2^*
Covariate		
Teacher Age	.10	1.10
Predictors		
Stressors	−.03	.86
Mental Health	.43**	13.90
Race (Black = 1, White = 2)	−.78**	11.53
Confidence in System-Level Supports	1.27**	97.22

*Note*. Model was fit using ordinal regression with robust standard errors for the teacher efficacy outcome variable. *p < .05, **p < .01.

### Research Question 3: What Resources do Teachers Believe They Need to Support Their Students?

**Figure 1.**
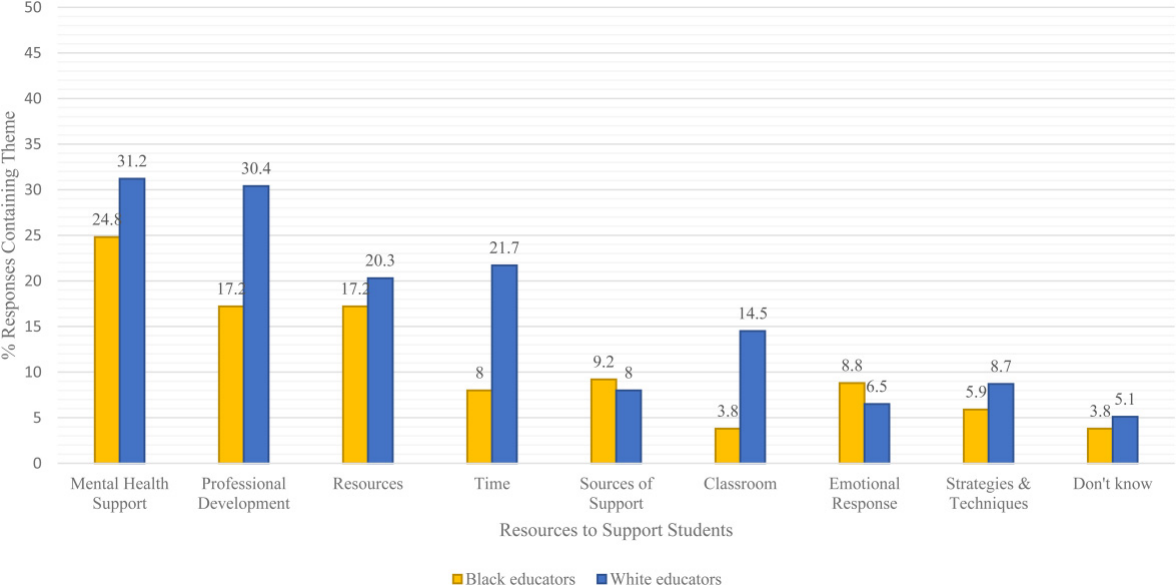
Qualitative themes by teacher's race on the resources needed to support students upon school reopening.

Our findings also revealed that teachers identified various forms of support to engage students further and improve self-efficacy. About 82.4% (*n* = 310) of the sample responded to what resources they would need to address the stress and trauma of their students. These responses were coded into nine themes (Figure 1). A description of each theme can be found in Appendix A. The findings revealed that Black and White teachers expressed similar ideals of the best ways to support students, with their responses falling into the following top three themes, (1) mental health support, (2) professional development, and (3) in-class resources.

Surveyed respondents indicated that the best way to assist students in transitioning to school was through mental health supports, with a quarter (24.8%) of Black teachers and 31.2% of White teachers agreeing. Respondents defined *mental health supports* as the act of hiring or improving the presence of mental health professionals within their schools. These individuals represented psychologists, social workers, and counselors, according to teachers. One teacher suggested the role of a health professional would be to work with students’ transition to the new academic year. They stated, “Support for students may be needed like having a counseling time for them. [And] having a time for them to close out their last school year in a proper way.” Another teacher articulated that assistance with students’ mental health would need to come from all professionals within the school, “I would need support from our school counselor, social worker, my principal and co-workers.”

When teachers were asked to describe the types of support that would aid students to transition back to school, 24 respondents agreed that they or other teachers needed mental health support. Both White (25.6%) and Black respondents (22.0%) expressed a need to support teachers within the mental health theme. One sugested that their peers needed “to have counseling sessions to cope also.”

Similar to providing mental health support, 83 teachers also requested professional development trainings to provide relevant information on how to support students. Respondents believed trainings would help provide context around the pandemic, strategies on how to address the needs of their students, and lessons on identifying trauma amongst students. In some instances, teachers recognized the unique needs of their students living in under-resourced communities and called for trainings that represented the populations they served. A respondent stated, “[It is vital to have] professional developments (PD) with people that have worked with children and that are equal to the demographics of children we teach on the day to day. Having a PD from someone who doesn't know the demographics is pointless.” Furthermore, another teacher requested ongoing training to support the student population they serve.

The third most common theme highlighted by survey respondents related to in-class resources. Overall, respondents requested informational packets to distribute to students. One teacher stated, “[We will need] age-appropriate resources teachers can use with their students (e.g., pamphlets, videos, etc.) about the Coronavirus, or any other pandemic.” Both White (20.6%) and Black (18.1%) teachers described the need for COVID-19 related items to assist them when students returned in the fall. Teachers requested plans that addressed the cleanliness procedures and sterilization process for classrooms and insisted on enough antibacterial solution and Personal Protective Equipment (PPE) to keep all healthy. One teacher stated, “Cleaning supplies and materials to demonstrate precautions [on] not spreading viruses. For example, [we need] wipes and time dedicated to washing hands, wiping keyboards, and at the same time not wasting materials and creating more garbage.”

In addition to COVID-19 related items, teachers appealed for technology, curriculum, and tangible content. Another teacher specified, “More socially emotional resources!!! Books, games, lessons…I’d love to have something that is more tailored to this specific situation.”

### Differences Between Groups

The literature shows a connection between teacher self-efficacy and the racial congruency of students and educators. So far, our findings show that Black teachers are more likely to be confident as they return to the classroom compared to their White peers. We also found differences in how Black and White teachers identified necessary supports for their students. The greatest gap between teachers emerged in the rate at which teachers spoke about the theme time. We found that White teachers (21.7%) were more likely to address time and transitions as a mechanism to support students as compared to their Black peers (8.0%). Based on respondents’ interpretations, we defined *time* as modified schedules and transitional periods for students to recover from COVID-19. Teachers wanted time to acknowledge the pandemic and the extent to which it affected students and their families. Without this initial step, respondents believed that students would be unable to return to their coursework. One White teacher stated, “[We should] not simply act as though nothing has happened and be sensitive to the differing transition processes of students, but how to also jumpstart a healthy academic routine and procedures.” A Black teacher reflected similar thoughts, “I would like to have social workers available to address these needs [emotional support for students]. I would also like time to build into the schedule to meet these particular concerns.”

Another striking difference emerged in the rate of responses between Black and White teachers around the topic of academics. Black teachers (3.8%) were less likely to address academics than their White peers (14.5%). Based on teachers’ responses, we defined *academics* as the pedagogical activity within the classroom. Results showed that respondents expressed concern about pushing ahead in the curriculum. Specifically, topics of concern included academic content, learning strategies, and classroom size, which could exacerbate learning disruptions. One White teacher expressed, “We need a major focus on wellness, and I am worried that we will immediately focus on lost academic time, to the detriment of people's well-being.” Similarly, one Black teacher stated,

I believe that being realistic about where students are academically after this extended break is going to be key in meeting the needs of students next school year. Mentally, students may have checked out as well. How to balance where students are academically and mentally in August will be the biggest struggle.

### Assessing Differences by Self-Efficacy

The authors were interested in exploring the ways teachers responded to questions given their different levels of self-efficacy to address the needs of their students. To assess, the authors compared qualitative responses between teachers who were least and most self-efficacious in addressing stress and trauma in students. The data showed that only Black teachers had enough respondents for comparison, with 35 indicating they felt least efficacious and 52 most efficacious. Only 34 White teachers identified as having low self-efficacy compared to only one person who expressed high self-efficacy. Given this difference, the authors only included differences in self-efficacy for Black teachers.

We found that Black teachers who felt least efficacious were more likely to request professional development trainings and less likely to request support from personnel (excluding mental health personnel) than their more confident Black peers. Roughly 20.0% of Black respondents who had the lowest self-efficacy levels requested professional development trainings compared to 3.8% of those who had the highest levels. In contrast, 21.2% of Black respondents who felt most confident suggested receiving support from school and county personnel. At the same time, not one teacher who identified as being the least efficacious requested support from these individuals.

## Discussion

Knowing that both teachers and students have returned to school under the weight of unprecedented COVID-19-related stressors, the current study aimed to understand teachers’ sense of self-efficacy to address the social and emotional needs of their students. Drawing from the literature examining important influences on teacher self-efficacy and racial congruency, we utilized data from a needs assessment of New Orleans public school teachers to identify personal and school factors associated with emotional support efficacy. Specifically, we examined the influence of teacher race, COVID-19 stressors, mental health, and confidence in system-level supports on teacher self-efficacy.

Although COVID-19-related stressors were negatively correlated with teacher self-efficacy, they were not a significant predictor of self-efficacy when all other factors were taken into account. The experience of stressors may be less critical for teacher self-efficacy than the impact those stressors have on teacher mental health (Jennings & Greenberg, 2009). In the current sample, COVID-19 related stressors were negatively correlated with teacher mental health, which did emerge as a significant predictor of teacher self-efficacy when all other factors were taken into account. Frequent emotional distress can reduce feelings of self-efficacy, particularly in teachers’ ability to connect with their students and provide social-emotional support (Jennings & Greenberg, 2009).

Teacher race was also associated with self-efficacy; Black teachers reported greater efficacy in addressing their students’ social and emotional needs than White teachers. Whereas Black teachers may derive a greater sense of efficacy due to their increased understanding of the cultural context of Black students’ lives (Lindsay & Hart, 2017; Milner, 2006), the efficacy of White teachers may be challenged by the lack of a culturally shared knowledge base (Bentley-Edwards et al., 2020). Interestingly, the heightened awareness brought on by the pandemic of the stressors their students typically face at home (Baker et al., 2021; Gewertz, 2020) could also have a differential impact on the efficacy of Black and White teachers. Similar findings were noted in the Gilliam et al. (2016) study that highlighted that teachers who have a greater sense of empathy were racially congruent to their students.

In addition to personal factors, teacher perceptions of the school context are critical for teacher self-efficacy (Hoy & Woolfolk, 1993). Contextual resources that ease job demands and help teachers achieve success promote teacher self-efficacy and mental health (Hoy & Woolfolk, 1993). We found that one type of contextual resource, perceived school responsiveness in addressing teachers’ own needs stemming from pandemic-related stress and trauma, was related to teacher self-efficacy in providing emotional support to their students. Our qualitative analyses provided insights into specific types of resources they viewed as responsive to their needs.

Overall, both Black and White teachers agreed that the top three resources needed to support students and teachers once they return to school are mental health supports, professional development, and in-class resources. White teachers spoke of time and creating a space where students could successfully transition from home to school. Qualitatively, we found the greatest differences in strategies to support students and teachers based on self-efficacy among Black teachers. Black respondents with low self-efficacy were more likely to request professional development and less likely to ask for support from the administration than their high self-efficacious Black peers.

In general, teachers need to feel confident in educating students upon returning to school, regardless of race. In addition, the researchers find that having high self-efficacy as a teacher is vital, especially during a pandemic. In this case, Black and White teachers described similar tools to promote self-efficacy. Our greatest difference among Black teachers by confidence level shows that those with high confidence were more likely to request support from their peers and leaders than their colleagues who exhibited low confidence. Additionally, those teachers with low self-efficacy may be more willing to use trainings to address their students’ emotional needs. These findings show the importance of assessing and addressing teachers’ self-confidence before classes begin. A clear finding that aligns with previous work (Hoy et al., 1990) is that an efficacious teacher is more likely to depend on their peers and administrator's support than one who is less confident.

### Limitations and Future Directions

The current study used data gathered as part of a district-wide needs assessment. The benefits of utilizing real-time data during a pandemic come with methodological limitations. First, because the survey was designed to assess teacher experiences in the most efficient way possible, single-item indicators were used to measure teacher self-efficacy, mental health, and confidence in system-level supports. Furthermore, the measurement of COVID-19-related stressors relied on items from a newly established measure. Although research in the fields of general health and well-being in the workplace have demonstrated the reliability and validity of single-item measures (Fisher et al., 2016), this work is still emerging as it relates to single-item indicators of global mental health, particularly among teachers (Baker et al., 2021; Eddy et al., 2020). Therefore, the current findings should be interpreted with caution, and future research is needed to establish the psychometric properties of the measures used in the current study.

A second limitation related to measurement in the current study is the operationalization of racial congruence between teachers and students. School racial composition has been used in prior research to define teacher-student racial congruence; studies have determined majority representation using cutoffs ranging from 40% to 75% of a single racial demographic comprising the school population (Martinez, 2020; McCarthy et al., 2020). The New Orleans public charter school population exceeds previously used cutoffs, as 81% of the student body is Black. However, unlike previous studies, we applied that cutoff at a district level, not at the level of individual schools. Although the vast majority of public schools in New Orleans enroll Black students at rates at or above 75% of the student population, about 5% of public schools enroll Black students at rates at or above 40% (New Orleans Education Equity Index, 2017). Therefore, our operationalization of racial congruence based on student racial demographics is consistent with previous research even as applied at the district level. Future research could ask teachers to explicitly or objectively document racial congruence between them and their students to determine self-efficacy based on classroom demographics. Furthermore, future research should examine racial congruence across a broader spectrum of racial and ethnic identities of teachers and the student bodies they serve. Aside from the racial categories of Black and White, no other racial or ethnic group comprised more than 5% of the sample, limiting our ability to examine how racial congruence relates to teacher self-efficacy in non-Black teachers of color.

Future research could also move beyond a focus on racial congruence to include discussions around teachers’ use of culturally sustaining pedagogy in racially congruent and noncongruent classrooms. Paris (2012) and Ladson-Billings (2014) have described the transition from culturally relevant pedagogy to culturally sustaining pedagogy, where teachers are called to engage multi-ethnic students in upholding cultural competence and providing access to dominant norms. Their work encourages teachers to not depend on a “static conception of what it means to be culturally relevant” (Ladson-Billings, 2014, p.77) but to “meet both demands [culturally sustaining pedagogies and student-driven learning] without diminishing either” (p.84). Future studies should assess the teachers’ use of culturally sustaining pedagogies in racially congruent and incongruent contexts to determine whether such pedagogies serve as a mechanism through which racial congruence influences teacher self-efficacy.

Our sample composition represents a third study limitation. The sample is drawn exclusively from public charter schools within a single district, and not all schools are equally represented in the sample. Given that few studies have investigated challenges to teachers’ professional functioning during the pandemic, we aimed to provide a timely and descriptive first look at an essential phenomenon in teachers. However, school-level variability may contribute meaningfully to the study findings and should be investigated in future work. Similarly, although our findings aligned well with the results of other teacher surveys during the pandemic (Cipriano & Brackett, 2020) and prior research on teacher self-efficacy, they are most representative of charter school teachers in a low-resource, high-poverty urban district. Caution should be used when generalizing these findings to dissimilar schools, such as those in traditional or rural districts. Our relatively small sample of teachers represents New Orleans teachers but not the overall teacher population; thus, generalizations about the differences in self-efficacy for Black and White teachers are not recommended.

A final limitation is that the data were collected at a single time point towards the beginning of the pandemic. Teachers were asked to look toward the future when schools reopened and rate their sense of efficacy in addressing the pandemic-related social-emotional needs of their students. Although their ratings were anticipatory, research has shown that ratings of teacher self-efficacy are based in part on an assessment of past performance (Tschannen-Moran & Woolfolk Hoy, 2007) and are stable over time (Lazarides et al., 2020), even for novice teachers. However, the extent to which teachers’ self-efficacy alters through a pandemic is unclear. Given that the survey was administered at the onset of the pandemic, it is possible that their confidence improves or regresses as the pandemic continues. The pattern of results observed in the current study may change once teachers return to the classroom, where other sources of self-efficacy beliefs can come into play, including feedback from others regarding their performance in managing student social-emotional needs and psychological and emotional arousal experienced back on the job (Tschannen-Moran & Woolfolk Hoy, 2007).

### Implications

Our results suggest that for schools to support an effective transition from remote to face-to-face learning, teachers need mental health supports for students and themselves. Administrators should consider creating spaces within the school building for teachers to solicit emotional support for professional or personal needs. Although not mentioned as frequently, teachers also requested that school administrators strengthen the professional role of teachers by providing relevant professional development, in-class resources such as technology and PPE, as well as effective plans to modify the curriculum to meet the emotional needs of students. However, building up the professionalism of teachers is not enough.

We also found that Black teachers expressed higher confidence in supporting their students than their White peers. Our results showed that Black respondents were more likely to address emotional support and were open about supporting the whole child during this pandemic. Researchers and teachers address the “invisible tax” teachers of color pay as they exert additional duties and mental health services to support students of color (Albert Shanker Institute, 2015). This tax can also be seen in Pabon’s (2016) work on her analysis of Black male teachers’ narratives on their experiences teaching in urban school settings. Her findings revealed that the teachers were deemed saviors of such environments but were not compensated for their efforts. Ultimately, the authors recommend creating spaces that uplift, honor, and draw from Black teachers and other teachers of color as experts.

Next, efforts are needed to increase the efficacy of White teachers in working with racially and culturally diverse students, which is consistent with the requests of a few White teachers that addressed cultural competence around formal training. School administrators could create additional spaces where teachers could 1) share their thoughts about the transition and provide emotional support to each other and 2) attend trainings that dive deep into anti-racism, cultural competence, and justice pedagogy. Siwatu et al. (2011) offered several self-efficacy-building activities that can be used to prepare teachers for racial and cultural incongruencies in the classroom. Another study by Tucker et al. (2005) to review, showed positive outcomes of a teacher training program that increased efficacy in working with culturally diverse students.

Lastly, teachers must be supported through significant disruptions, especially with an unknown end date for the pandemic and future growing numbers of natural disasters. Unfortunately, research on disasters and schools shows that teachers are often the first responders and responsible for supporting the mental health of their students and communities (Davis et al., 2021). Schools must transition from solely relying on teachers’ emotional labor to properly equip pedogeological spaces with professionals who can meet the needs of traumatized students and teachers. With mental health professionals, teachers can focus on meeting the academic needs of their students and help with resuming schooling to normal.

## Conclusion

As indicated earlier, Hurricane Katrina created a massive overhaul of schooling for the students and teachers in New Orleans. Since that disaster, teachers across the city had to rethink education for its most socially and historically marginalized students. Over a decade later, the city is faced with a new disaster that affects how teachers shape schooling. This study shows that teachers are coming to their classrooms already acknowledging the pain and trauma of COVID-19 and are prepared to identify the resources needed to meet mental health needs. Their preemptive responses will likely improve the self-efficacy of struggling teachers and ultimately care for students who are amidst a pandemic.

To date, little work investigates the impact of a pandemic on teachers’ self-efficacy. Unfortunately, schools are functioning in a prolonged pandemic alongside repeated natural disasters. Educational institutions must adapt to the changing landscape and learn to maintain or improve the self-efficacy of teachers, especially during major educational disruptions. This paper adds to the existing body of work on self-efficacy by shedding light on the extent teachers feel efficacious in their ability to address the whole child, the factors that influence their self-confidence, and the types of resources that will support students’ recovery, all during a worldwide pandemic. Similar to prior work, our results show that it is imperative for teachers to feel confident in their ability to help students survive and thrive in tumultuous times.

## Appendix A

### Definitions of the Nine Qualitative Themes

1. Mental Health Support – Details about receiving support geared toward mental health, including who receives it and what support resembles.
2. Professional Development – Access to professional development trainings and webinars which addressed topics like schooling, mental health, or the COVID-19 pandemic.
3. In-class Resources – Respondents described resources or referenced who should receive the resource. Resources were items related to technology, human resources (e.g., hiring extra school personnel, excluding mental health personnel), and PPE. Respondents identified individuals such as students, their families, teachers, and themselves, as possible recipients of the resources.
4. Time – the transition from virtual to face-to-face instruction.
5. Sources of Support – Individuals that support students and teachers in school. Individuals represented the school nurse, students’ families, educators, and administrators.
6. Classroom – All things that occur in the classroom with a focus on the academic success of the child. This includes curriculum development.
7. Emotional Support – An emotional response to students transitioning to school. Responses represented codes such as compassion, patience, positive attitude, trust, faith, love, peace, doubt, and safety.
8. Strategies & Techniques – A strategy or plan for when students returned to school.
9. Don't Know – Respondents were unsure of how to answer the question.

## References

[bibr1-00420859221099834] Ahmad F. Jhajj A. Stewart D. Burghardt M. Bierman A. S. (2014). Single item measures of self-related mental health: A scoping review. BMC Health Service Research, 14(1), 398. 10.1186/1472-6963-14-398 PMC4177165 25231576

[bibr2-00420859221099834] Albert Shanker Institute. (2015). *The state of teacher diversity in American education*. https://www.shankerinstitute.org/resource/state-teacher-diversity-executive-summary

[bibr3-00420859221099834] Babineau K. Karapetyan A. Rossmeier V. (2020). The state of public education in New Orleans. Cowen Institute. https://files.eric.ed.gov/fulltext/ED607281.pdf.

[bibr4-00420859221099834] Baker C. N. Peele H. Daniels M. Saybe M. Whalen K., Overstreet K., & the New Orleans Trauma-Informed Schools Learning Collaborative. (2021). The experience of COVID-19 and its impact on teachers’ mental health, coping, and teaching. School Psychology Review, 50(4), 491‐504. 10.1080/2372966X.2020.1855473

[bibr5-00420859221099834] Bandura A. (1997). Self-efficacy: The exercise of control. W. H. Freeman and Company.

[bibr6-00420859221099834] Barrett E. J. Barron Ausbrooks C. Y. Martinez-Cosio M. (2012). The tempering effect of schools on students experiencing a life-changing event: Teenagers and the hurricane katrina evacuation. Urban Education, 47(1), 7‐31. 10.1177/0042085911416011

[bibr7-00420859221099834] Bentley-Edwards K. L. Stevenson H. C. Thomas D. E. Adams-Bass V. N. Coleman-King C. (2020). Academic affect shapes the relationship between racial discrimination and longitudinal college attitudes. Social Psychology of Education, 23(5), 1233‐1257. 10.1007/s11218-020-09578-8 PMC9977137 36865586

[bibr8-00420859221099834] Bettini E. Park Y. (2021). Novice teachers’ experiences in high-poverty schools: An integrative literature review. Urban Education, 56(1), 3‐31. 10.1177/0042085916685763

[bibr9-00420859221099834] Bixler D. Miller A. D. Mattison C. P. Taylor B. Komatsu K. Peterson Pompa X. Moon S. Karmarkar E. Liu C. Y. Openshaw J. J. Plotzker R. E. Rosen H. E. Alden N. Kawasaki B. Siniscalchi A. Leapley A. Drenzek C. Tobin-D’Angelo M. Kauerauf J., … X. Lin (2020). SARS-CoV-2–Associated deaths among persons aged<21 years - United States, February 12-July 31, 2020. MMWR. Morbidity and Mortality Weekly Report, 69(37), 1324‐1329. 10.15585/mmwr.mm6937e4 32941417

[bibr10-00420859221099834] Cannon S. R. Davis C. R. Fuller S. C. (2020). Preparing for the next natural disaster: Understanding how hurricanes affect teachers and schooling. AASA Journal of Scholarship & Practice, 17(2), 6‐15. https://aasa.org/uploadedFiles/Publications/JSPSummer2020.FINAL.v2.pdf#page=6

[bibr11-00420859221099834] Cipriano C. Brackett. (2020, June 15). Teachers are anxious and overwhelmed. They need SEL now more than ever. *EdSurge News*. https://www.edsurge.com/news/2020-04-07-teachers-are-anxious-and-overwhelmed-they-need-sel-now-more-than-ever.

[bibr12-00420859221099834] Daniel M. E. Harwell D. (2010). Preschool teachers’ perceptions of self-efficacy in terms of meeting the needs of students following a critical incident (The example of Hurricane Katrina). e-International Journal of Educational Research, 1(1), 77‐87. http://www.e-ijer.com/en/download/articlefile/89706

[bibr13-00420859221099834] Davis C. R. Cannon S. R. Fuller S. C. (2021). The storm after the storm: The long-term lingering impacts of hurricanes on schools. Disaster Prevention and Management, 30(3), 264‐278. 10.1108/DPM-03-2020-0055

[bibr14-00420859221099834] Dixson A. Dingus J. (2008). In search of our mothers’ gardens: Black women teachers and professional socialization. Teachers College Record, 110(4), 805‐837. 10.1177/016146810811000403

[bibr15-00420859221099834] Donnellan M. B. Oswald F. L. Baird B. M. Lucas R. E. (2006). The Mini-IPIP Scales: Tiny-yet-effective measures of the Big Five Factors of Personality. Psychological Assessment, 18(2), 192‐203. 10.1037/1040-3590.18.2.192 16768595

[bibr16-00420859221099834] Eddy K. C. Herman W. M. Reinke C. L. (2020). Advances in understanding and intervening in teacher stress and coping. Journal of School Psychology, 78, 69‐74. 10.1016/j.jsp.2020.01.001 32178812

[bibr17-00420859221099834] Fisher G. G. Mattews R. A. Gibbons A. M. (2016). Developing and integrating the use of single-item measures in organizational research. Journal of Occupational Health Psychology, 21(1), 3‐23. 10.1037/a0039139 25894198

[bibr18-00420859221099834] Geerlings J. Thijs J. Verkuyten M. (2018). Teaching in ethnically diverse classrooms: Examining individual differences in teacher self-efficacy. Journal of School Psychology, 67, 134‐147. 10.1016/j.jsp.2017.12.001 29571529

[bibr19-00420859221099834] Gewertz C. (2020, April 20). Exhausted and grieving: Teaching during the coronavirus crisis. *The Miami Times*. https://www.miamitimesonline.com/COVID-19_hub/exhausted-and-grieving-teaching-during-the-coronavirus-crisis/article_d494c94c-8357-11ea-bcb7-075cf66f7c06.html

[bibr20-00420859221099834] Gilliam W. S. Maupin A. Reyes C. R. Accavitti M. Shic F. (2016). Do early teachers’ implicit biases regarding sex and race relate to behavior expectations and recommendations of preschool expulsions and suspensions? Yale Child Study Center. https://medicine.yale.edu/childstudy/zigler/publications/Preschool%20Implicit%20Bias%20Policy%20Brief_final_9_26_276766_5379_v1.pdf.

[bibr22-00420859221099834] Grasso D. J. Briggs-Gowan M. J. Ford J. D. Carter A. S. (2020). *The Epidemic – Pandemic Impacts Inventory (EPII)*. https://www.phenxtoolkit.org/toolkit_content/PDF/Grasso_EPII.pdf

[bibr23-00420859221099834] Greene D. (2020). “Black female teachers are our school parents!!”: Academic othermothering depicted in multicultural young adult texts. Journal of Language & Literacy Education, 16(1), 1‐19. https://files.eric.ed.gov/fulltext/EJ1253928.pdf

[bibr25-00420859221099834] Hamilton L. S. Kaufman J. H. Diliberti M. K. (2020). Teaching and leading through a pandemic: Key findings from the American educator panels spring 2020 COVID-19 surveys. RAND Corporation. https://www.rand.org/pubs/research_reports/RRA168-2.html

[bibr26-00420859221099834] Herman K. C. Sebastian J. Reinke W. M. Huang F. L. (2021). Individual and school predictors of teacher stress, coping, and wellness during the COVID-19 pandemic. School Psychology, 36(6), 483‐493. 10.1037/spq0000456 34766812

[bibr27-00420859221099834] Hoerger M. (2010). Participant dropout as a function of survey length in internet-mediated university studies: Implications for study design and voluntary participation in psychological research. Cyberpsychology Behavioral Social Network, 13(6), 697‐700. 10.1089/cyber.2009.0445 PMC4367493 21142995

[bibr28-00420859221099834] Hoff R. A. Bruce M. L. Kasl S. V. Jacobs S. C. (1997). Subjective ratings of emotional health as a risk factor for major depression in a community sample. The British Journal of Psychiatry, 170, 167‐172. 10.1192/bjp.170.2.167 9093508

[bibr30-00420859221099834] Hoy W. K. Tarter T. C. J. Bliss J. R. (1990). Organizational-climate, school-health, and effectiveness: A comparative-analysis. Educational Administration Quarterly, 26, 260‐279. 10.1177/0013161X90026003004

[bibr31-00420859221099834] Hoy W. K. Woolfolk A. E. (1993). Teachers’ sense of efficacy and the organizational health of schools. The Elementary School Journal, 93(4), 355‐372. 10.1086/461729

[bibr33-00420859221099834] Jennings P. A. Greenberg M. T. (2009). The prosocial classroom: Teacher social and emotional competence in relation to student and classroom outcomes. Review of Educational Research, 79(1), 491‐525. 10.3102/0034654308325693

[bibr34-00420859221099834] Kunemund R. L. McCullough S. N. Williamsn C. D. Miller C. C. Sutherland K. S. Conroy M. A. Granger K. (2020). The mediating role of teacher self-efficacy in the relation between teacher-child race mismatch and conflict. Psychology in the Schools, 57(11), 1757‐1770. 10.1002/pits.22419

[bibr35-00420859221099834] Ladson-Billings G. (1994). What we can learn from multicultural education research. Educating for Diversity, 51(8), 22‐26. https://www.ascd.org/el/articles/what-we-can-learn-frommulticultural-education-research

[bibr36-00420859221099834] Ladson-Billings G. (2014). Culturally relevant pedagogy 2.0: A.k.a. The remix. Harvard Educational Review, 84(1), 74‐84. 10.17763/haer.84.1.p2rj131485484751

[bibr37-00420859221099834] Lazarides R. Watt H. M. G. Richardson P. W. (2020). Teachers’ classroom management self-efficacy, perceived classroom management and teaching contexts from beginning until mid-career. Learning and Instruction, 69, 101346. 10.1016/j.learninstruc.2020.101346

[bibr38-00420859221099834] Lincove J. A. Barrett N. Strunk K. O. (2018). Lessons from Hurricane Katrina: The employment effects of mass dismissal of New Orleans teachers. Educational Researcher, 47(3), 191‐203. 10.3102/0013189X18759542

[bibr39-00420859221099834] Lindsay C. A. Hart C. M. D. (2017). Exposure to same-race teachers and student disciplinary outcomes for black students in North Carolina. Education Evaluation and Policy Analysis, 39(3), 485‐510. 10.3102/0162373717693109

[bibr40-00420859221099834] Martinez M. J. (2020). Does school racial composition matter to teachers: Examining racial differences in teachers’ perceptions of student problems. Urban Education, 55(7), 992‐1020. 10.1177/0042085918770709

[bibr41-00420859221099834] McCarthy C. J. Dillard J. Fitchett P. G. Boyle L. Lambert R. G. (2020). Associations between teacher–student racial/ethnic congruence and public school teachers’ risk for stress. Urban Education, 1‐28. 10.1177/0042085919894049

[bibr42-00420859221099834] Miles M. B. Huberman A. M. (1994). Qualitative data analysis (2nd ed.). Sage.

[bibr43-00420859221099834] Milner H. R. (2006). The promise of Black teachers’ success with Black students. Educational Foundations, 20(3), 89‐104. https://files.eric.ed.gov/fulltext/EJ794734.pdf

[bibr44-00420859221099834] Milner H. R. (2012). But what is urban education? Urban Education, 47(3), 556‐556. 10.1177/0042085912447516

[bibr45-00420859221099834] Milner H. R. (2013). Analyzing poverty, learning, and teaching through a Critical Race Theory lens. Review of Research in Education, 37(1), 1‐53. 10.3102/0091732X12459720

[bibr46-00420859221099834] Milner H. R. Woolfolk Hoy A. (2003). A case study of an African American teacher’s self-efficacy, stereotype threat, and persistence. Teaching and Teacher Education, 19(2), 263‐276. 10.1016/S0742-051X(02)00099-9

[bibr47-00420859221099834] Modan N. (2020, July 8). *Pitting mental health against safety, national leaders point to SEL in school reopening debate*. K-12 Dive. https://www.k12dive.com/news/pitting-mental-health-against-safety-national-leaders-point-to-sel-in-scho/581159/

[bibr48-00420859221099834] Muhammad G. E. Dunmeyer A. Starks F. D. Sealey-Ruiz Y. (2020). Historical voices for contemporary times: Learning from black women educational theorists to redesign teaching and teacher education. Theory into Practice, 59(4), 419‐428. 10.1080/00405841.2020.1773185

[bibr49-00420859221099834] New Orleans Education Equity Index. (2017). *Equity matters: A look at educational equity in New Orleans public schools*. New Orleans Education Equity Index. http://neworleansequityindex.org/files/downloads/New-Orleans-Education-Equity-Index-Report-2017.pdf

[bibr50-00420859221099834] National Oceanic and Atmospheric Administration (NOAA). (n.d.). *Coastal fast facts*. https://coast.noaa.gov/data/nationalfacts/pdf/hand-out-coastal-fast-facts.pdf

[bibr51-00420859221099834] Pabon A. (2016). Waiting for Black superman: A look at a problematic assumption. Urban Education, 51(8), 915‐939. 10.1177/0042085914553673

[bibr52-00420859221099834] Palinkas L. A. Aarons G. A. Horowitz S. Chamberlain P. Hurlburt M. Landsverk J. (2011). Mixed method designs in implementation research. Administration and Policy in Mental Health, 38(1), 44‐53. 10.1007/s10488-010-0314-z 20967495 PMC3025112

[bibr53-00420859221099834] Paris D. (2012). Culturally sustaining pedagogy: A needed change in the stance, terminology, and practice. Educational Researcher, 41(3), 93‐97. 10.3102/0013189X12441244

[bibr54-00420859221099834] Paris D. Alim H. S. (2017). Culturally sustaining pedagogies: Teaching and learning for justice in a changing world. Teachers College Press.

[bibr56-00420859221099834] Pollack T. M. (2013). Unpacking everyday “teacher talk” about students and families of color: Implications for teacher and school leader development. Urban Education, 48(6), 863‐894. 10.1177/0042085912457789

[bibr57-00420859221099834] Seyle D. C. Widyatmoko C. S. Silver R. C. (2013). Coping with natural disasters in Yogyakarta, Indonesia: A study of elementary school teachers. School Psychology International, 34(4), 387‐404. 10.1177/0143034312446889

[bibr58-00420859221099834] Silverman H. (2020, March 24). Louisiana governor says his state has the fastest growth rate of coronavirus cases in the world. *CNN*. https://www.cnn.com/2020/03/23/us/louisiana-coronavirus-fastest-growth/index.html.

[bibr59-00420859221099834] Siwatu K. O. Frazier P. Osaghae O. J. Starker T. V. (2011). From maybe I can to yes I can: Developing pre-service and inservice teachers’ self-efficacy to teach African American students. The Journal of Negro Education, 80(3), 209‐222. 10.1016/j.tate.2010.09.004

[bibr60-00420859221099834] Skidmore M. Lim J. (2020). *Natural disasters and their impact on cities*. Oxford Bibliographies. https://www.oxfordbibliographies.com/view/document/obo-9780190922481/obo-9780190922481-0014.xml

[bibr62-00420859221099834] Sosa T. Gomez K. (2012). Connecting teacher efficacy beliefs in promoting resilience to support of Latino students. Urban Education, 47(5), 876‐909. 10.1177/0042085912446033

[bibr63-00420859221099834] Strauss J. Corbin A. (1990). Grounded theory research: Procedures, canons, and evaluative criteria. Qualitative Sociology, 19(1), 418‐427. 10.1515/zfsoz-1990-0602

[bibr64-00420859221099834] Tschannen-Moran M. Woolfolk Hoy A. (2001). Teacher efficacy: Capturing an elusive construct. Teaching and Teacher Education, 17(7), 783‐805. 10.1016/S0742-051X(01)00036-1

[bibr65-00420859221099834] Tschannen-Moran M. Woolfolk Hoy A. (2007). The differential antecedents of self-efficacy beliefs of novice and experienced teachers. Teaching and Teacher Education, 23(6), 944‐956. 10.1016/j.tate.2006.05.003

[bibr66-00420859221099834] Tucker C. M. Porter T. Reinke W. M. Herman K. C. Ivery P. D. Mack C. E. Jackson E. S. (2005). Promoting teacher efficacy for working with culturally diverse students. Preventing School Failure: Alternative Education for Children and Youth, 50(1), 29‐34. 10.3200/PSFL.50.1.29-34

[bibr67-00420859221099834] U.S. Department of Education. (2021). *Education in a pandemic: The disparate impacts of COVID-19 on America‘s students*. https://www2.ed.gov/about/offices/list/ocr/docs/20210608-impacts-of-covid19.pdf

[bibr68-00420859221099834] Villegas A. M. Irvine J. J. (2010). Diversifying the teaching force: An examination of major arguments. Urban Review, 42(3), 175‐192. 10.1007/s11256-010-0150-1

[bibr69-00420859221099834] Warren C. A. (2015). Conceptions of empathy and the work of good-intentioned early career white female teachers. Urban Education, 50(5), 572‐600. 10.1177/0042085914525790

[bibr70-00420859221099834] Williams G. M. (2012). Developing short, practical measures of well-being. In Anderson M. (Ed.), Contemporary ergonomics and human factors (pp. 203‐210). Taylor & Francis. 10.1201/b11933-52

[bibr71-00420859221099834] Williams G. M. Smith A. P. (2016). Using single-item measures to examine the relationships between work, personality, and well-being in the workplace. Psychology (Savannah, Ga ), 7(6), 753‐767. 10.4236/psych.2016.76078

[bibr72-00420859221099834] Xu W. Hou Y. Hung Y. S. Zou Y. (2013). A comparative analysis of spearman’s rho and kendall’s tau in normal and contaminated normal models. Signal Processing, 93(1), 261‐276. 10.1016/j.sigpro.2012.08.005

[bibr73-00420859221099834] Zee M. Jong P. F. D. Koomen H. M. Y. (2016). Teachers’ self-efficacy in relation to individual students with a variety of social–emotional behaviors: A multilevel investigation. Journal of Educational Psychology, 108(7), 1013‐1027. 10.1037/edu0000106

